# Development and Validation of a Self-Assessment Tool for an Integrative Model of Health Promotion in Hospitals: Taiwan’s Experience

**DOI:** 10.3390/ijerph16111953

**Published:** 2019-06-01

**Authors:** Ying-Wei Wang, Shu-Li Chia, Chien-Ming Chou, Michael S. Chen, Jürgen M. Pelikan, Cordia Chu, Mei-Hsiu Wang, Chiachi Bonnie Lee

**Affiliations:** 1 Health Promotion Administration, Ministry of Health and Welfare, Taipei 10341, Taiwan; drywwang@gmail.com (Y.-W.W.); lih.chia@hpa.gov.tw (S.-L.C.); h90slamdown@hpa.gov.tw (C.-M.C.); 2 School of Medicine, Tzu Chi University, Hualien 97004, Taiwan; 3 Department of Healthcare Administration, Asia University, Taichung 41354, Taiwan; aping.chen@msa.hinet.net; 4 Department of Social Welfare, National Chung Cheng University, Chiayi 62102, Taiwan; 5 CC Collaborating Centre for Health Promotion in Hospitals and Health Care, Gesundheit Österreich GmbH (Austrian Public Health Institute), 1010 Vienna, Austria; juergen.pelikan@univie.ac.at; 6 Department of Sociology, University of Vienna, 1090 Vienna, Austria; 7 Center for Environment and Population Health, Griffith University, Brisbane 4111, Australia; c.chu@griffith.edu.au; 8 Department of Health Services Administration, China Medical University, Taichung 40402, Taiwan; u105075204@cmu.edu.tw

**Keywords:** validation, integrative model, health-promoting hospitals, Taiwan

## Abstract

The Health Promotion Administration of Taiwan launched an integrative certification initiative in 2016 to streamline a plural system of certifications of health promotion in hospitals. It endeavored to replace original certifications, thereby establishing the proposal of a self-assessment instrument to aid in this integration. This study aimed to verify the robustness of this self-assessment tool by conducting exploratory factor analyses through stratification, reliability tests, content and construct validity tests, and specialist evaluations, which were convened to judge the comprehensibility, applicability, and importance of the standards and measures of this tool. A stratified random sampling of 46 hospitals was performed to confirm the validity of this tool. The tool rendered a floor effect of 0% and a ceiling effect of 13%. A valid factor structure and internal consistency (α ranged from 0.88 to 0.96) in each standard were verified. Hospitals with previous certificates or with 300+ beds achieved high compliance scores. A majority of experts agreed that the sub-standards were comprehensible (≥80%), applicable (≥70%), and important (≥70%). Finally, we conclude that the self-assessment tool is valid and can serve as a reference for other countries with hospitals committed to health promotion in hospital settings.

## 1. Introduction

The health-promoting hospital (HPH) is a form of setting approach for health promotion initiated by the World Health Organization in 1988 [1]. In this setting, a hospital strives to “improve health gain for its stakeholders by developing structure, cultures, decisions and process” [2]. A WHO self-assessment tool for health promotion in hospitals was developed in 2001 to ensure the overall quality of health services [3,4]. The tool encompasses five standards (40 measures), namely, management policy, patient assessment, patient information and intervention, promoting a healthy workplace, and continuity and cooperation. After 10 years, the WHO Collaborating Center for Evidence-Based Health Promotion in Hospitals and Health Services updated the HPH standards and integrated clinical health promotion (CHP) of healthy lifestyles [5]. The updated five standards (46 measures) included policy and leadership, patient assessment, patient information and intervention, promoting a healthy workplace and ensuring capacity for CHP, and implementation and monitoring.

Subsequently, task forces or working groups of the international HPH network have been established to improve health gains for patients, staff, and communities by organization-wide integration of health promotion under the headings of HPH and Environment since 2010, HPH and Age-Friendly Health Care since 2011, HPH and Patient- and Family-Engaged Health Care since 2016, and HPH and Health-Literate Health Care Organizations (HPH and HLO) since 2016 [6,7]. Furthermore, in accordance with the ideals of HPH, the international HPH network works together with the Global Network for Tobacco-Free Healthcare Services; with eight standards, the latter aims to implement and sustain effective tobacco management and cessation policies within healthcare services via a systematic approach [8].

In Taiwan, hospitals are suitable settings in which to implement health promotion among patients, staff, and communities as well as build low-carbon environments. Hospitals in Taiwan could reach a vast population, as seen in how hospitals consumed 45.6% of national health expenditure in 2017 (20.9% for inpatient and 24.7% for outpatient services) [9]. Despite the existence of a referral system, patients in Taiwan tend to visit hospitals directly without a referral. Similarly to numbers worldwide, over 50% of the causes of deaths in Taiwan were non-communicable diseases in 2017 [10]. These were mostly related to behavioral risk factors, including harmful use of alcohol and tobacco, dietary behavior, and physical inactivity [11]. Patients with chronic conditions often require professional assistance to maintain a healthy lifestyle and adhere to complex medication and nutrition regimens [2]. Furthermore, Taiwan became an aged society in 2018 and will probably take only eight years to become a super-aged society with more than 20% of the population aged over 65 years old by 2026 [12]. People aged 65 and over used 36.9% of the medical services under national health insurance in 2017 [13]. Establishing an age-friendly healthcare system is therefore imperative to meet the health needs of the country’s older population. The quality of care relies on healthy staff. However, research has found relatively high rates of occupational injuries, musculoskeletal disorders, and verbal or sexual harassment/violence among healthcare workers in Taiwan [14,15]. Furthermore, 98% of the energy in Taiwan is imported [16]. Hospitals accounted for 14.9% of energy consumption among the non-manufacturing industries [17]. Hospitals could save 6%–10% of energy if they exercise conservation practices in power, lighting, air conditioning, and transaction equipment [18].

The implementation of certifiable management systems (MSs) is considered a strategy via which to improve the quality of care by strengthening the structure of health organizations and processes of service delivery. A national HPH network was founded in Taiwan in 2006, which, by absolute number of member institutions, is the largest national network within the international HPH network. Taiwan’s Health Promotion Administration (HPA) has successively instituted a series of health promotion certifications in hospitals to ensure health promotion in hospitals; examples include the HPH in 2007 (148 out of 490 hospitals certificated by the end of 2016), environment-friendly healthcare in 2010 (172 hospitals certificated), age-friendly healthcare (169 hospitals certificated), and smoke-free healthcare (209 hospitals certificated) in 2011 [19]. These certifications were strewn with duplication and overlapping, however. For example, guidelines on how to identify the need for health promotion among groups of patients and information provided to the patients recorded in their medical records are required in HPH, age-friendly healthcare, and smoke-free hospitals; staff orientation that addresses the hospital’s health promotion policies and personnel and functions for the coordination of health promotion are required in all four of the above-mentioned certification systems. Moreover, certification/accreditation might burden the hospital staff with a heavy paperwork load [20]. Organizations might also face difficulties in simultaneously administering multiple MSs [21]. A review has demonstrated that the benefits obtained from an integrated approach are greater than those obtained from a single MS approach [22]. A streamlined MS that integrates quality, environmental friendliness, social responsibilities, and occupational health and safety is deemed a sensible strategy for organizational sustainability and competitiveness [23].

As a result, Taiwan’s HPA launched an integrative certification model and proposed a set of seven standard and 46 sub-standard self-assessment tools for the certification of the integration of HPH (hereafter iHPH) at the end of 2016. A self-assessment form is regarded as a useful instrument for the standardization of health promotion in hospitals [3,24]. A self-assessment iHPH tool was accordingly developed for this purpose by the HPA [25]. A panel of eight representatives from the areas of health policy; health services administration; and professional groups in healthcare, aging, and low-carbon management was set up to review the draft updated HPH standards as illustrated by Tønnesen and Svane [5]. The eight-member panel also reviewed the “old” WHO HPH standards [4] and the standards laid out in the Tobacco-Free Healthcare Services [8], Age-Friendly Health Care [26,27], and Environment-Friendly Hospital Initiative [28]. The iHPH standards further incorporated aspects of shared decision-making (SDM) as advocated by the New Haven Recommendations [29] and organizational health literacy [30]. The documentation review was concluded at the end of 2016 and a set of sub-standards nested in the original standards proposed after an expert validity test. Twenty-one hospitals were included to test the reliability of the tool and 10 other hospitals volunteered to undergo the pilot certification process verified by the eight-member panel. The iHPH self-assessment tool was then finalized on the basis of feedback from expert opinion surveys, expert consensus workshops, and satisfaction surveys from the hospitals that underwent pilot certification using the tool. 

We further investigated the validity of the tool with factor analysis to verify its robustness. Our aim was to ensure the factor structure of the seven standards, ceiling and floor effects, and internal consistency. In addition, we assessed the construct validity by analyzing the association between self-reported compliance scores and characteristics of a stratified sample of 46 hospitals. These hospitals also offered their ratings on the importance, comprehensibility, and applicability of the measurable elements.

## 2. Materials and Methods 

### 2.1. Description of the Self-Assessment Tool

The iHPH standards covered seven domains: (1) policy and leadership, (2) patient assessment, (3) patient information and intervention, (4) a healthy workplace and capacity for CHP, (5) implementation and monitoring, (6) age-friendly healthcare, and (7) environment-friendly healthcare [31]. Table 1 lists the sub-standards and the specific measures of the seven standards. Thirty-seven out of the 40 sub-standards of the old WHO HPH standards are covered in those of the iHPH standards. Measures regarding commitment to CHP, research and development in CHP, prohibition against accepting donations or sponsorships from tobacco vendors and sales of tobacco or e-cigarette products, SDM planning, favorable communication environments for patients and their family members, and plans for promoting health literacy were newly added. Furthermore, Standard 6 (age-friendly environments and provision of elderly services and plans) and Standard 7 (annual energy and water conservation, medical waste reduction, green procurement, and improvement plans) were appended. Sub-standards can be assessed as completely fulfilled, partly fulfilled, or not fulfilled. This three-level response format was selected with reference to the WHO standards of health promotion in hospitals [4]. With the exception of Standards 6 and 7, where sub-standards were composed of two to five items, “completely fulfilled” was defined as all items fulfilled; “partly fulfilled” was defined as not all items fulfilled but at least two items fulfilled; and “not fulfilled” was defined as none or only one item fulfilled. Sub-standards 6.1.1–6.2.4 and 7.1.1–7.1.4 were defined by the levels of fulfillment explicitly indicated in the manual [31].

### 2.2. Data Collection

We conducted a cross-sectional questionnaire survey with a stratified random sampling of 46 hospitals (a ratio of two iHPH to one non-iHPH match) from November to December 2018. The stratified random sampling considered iHPHs with or without certification, hospital levels (district hospitals, regional hospitals, and medical centers), and hospitals which were either general or not. We used Excel to compute random orders for 477 hospitals from different categories. Finally, 31 iHPHs and 15 non-iHPHs were included in the study. For iHPHs, representatives or coordinators for the iHPH certification or managers responsible for health promotion were invited to fill out the questionnaire. For non-iHPHs, managers responsible for health promotion were invited to fill out the questionnaire. Respondents were instructed to fill out the questionnaires after discussion with colleagues from other departments involved in health promotion. A blank space was provided in the questionnaire to accommodate open narrative opinions from the participants. A fee of US$65 was given to each respondent as compensation for their time and effort. Data collection was approved by the Research Ethics Committee of China Medical University Hospital (CMUH107-REC3-138).

### 2.3. Data Analysis

We computed compliance scores by rating each of the 46 measurable elements as not fulfilled (zero points), partly fulfilled (one point), and completely fulfilled (two points). We then summed them to obtain total scores for each standard and the overall standards. The seven standards and corresponding 46 measures of the iHPH self-assessment tool were identified in light of theory. We conducted exploratory factor analyses (EFA) by stratification with Kaiser-Meyer-Olkin (KMO) values, Bartlett’s test of sphericity, communalities, factor loadings, eigenvalues, and a scree test within each standard using principal axis factoring to detect the factor structure of the measures in each standard [32]. This approach has been used in a previous study [33]. We further evaluated the validity in light of the development of WHO HPH standards by following validation procedures of the major characteristic parameters [3]. We profiled the distribution of the overall compliance scores of the iHPHs and studied the floor and ceiling effects as a proportion of responses in the lowest and highest scores. To test construct validity, we investigated the associations between hospital characteristics and self-reported compliance scores by using the Mann-Whitney U Test, owing to the non-normal distributions of compliance scores. We further conducted reliability tests with Cronbach’s alpha to confirm internal consistency. Finally, we studied the levels of comprehensibility, applicability, and importance of each sub-standard with a five-point Likert scale ranging from one (strongly disagree) to five (strongly agree). The total score of each of the seven domains was converted to a figure on a scale of 10 for standard comparisons using a standardized scale. 

## 3. Results

Table 2 presents the characteristics of participating hospitals. The survey involved 12 public hospitals (26.1%), 16 private hospitals (34.8%), and 18 non-profit private hospitals (39.1%). Regarding the hospital levels, six were medical centers (13.0%), 18 were regional hospitals (39.1%), and 22 were district hospitals (47.8%). Fifty percent of hospitals owned less than 300 beds. More than half of the hospitals had been certificated as HPHs (54.3%), age-friendly healthcare hospitals (50.0%), smoke-free healthcare hospitals (71.7%), or environment-friendly hospitals (56.5%) before 2016.

### 3.1. Factory Analysis and Internal Consistency

According to the EFA results (Table 3), the KMO values were higher than 0.76; all items in each of the standards had factor loadings higher than 0.62 and every standard showed only one component as designed using an eigenvalue of 1 and scree tests. The proportions of explained variance in these seven standards were 71.9%, 60.5%, 70.2%, 67.0%, 75.6%, 55.8%, and 80.3%. The Cronbach’s alpha coefficients for each standard were very high, being 0.944 for policy and leadership, 0.878 for patient assessment, 0.961 for patient information and intervention, 0.887 for healthy workplace and capacity for CHP, 0.958 for implementation and monitoring, 0.896 for age-friendly healthcare, and 0.923 for environment-friendly healthcare, suggesting a high degree of reliability of the standards.

### 3.2. Content Validity

For content validity (Table 4), the overall mean value of compliance scores with the tool was 66.0 ± 29.5 (ranging from 0 to 92). The total assessment tool demonstrated an acceptable floor effect of 0% and a ceiling effect of 13%. For the single standards, mild floor effects were observed in Standard 5 (21.7%) and stronger ceiling effects were found in all of the seven standards (between 21.7% and 63.0%).

### 3.3. Construct Validity

For construct validity (Table 5), hospitals with previous experience as iHPHs, HPHs, international HPH network membership hospitals, age-friendly healthcare hospitals, environment-friendly healthcare hospitals, smoke-free hospitals, and those with more than 300 beds had significantly higher levels of compliance with the seven standards and overall compliance when compared with those without certifications or with less than 300 beds. The hospitals with the above-mentioned certifications also showed higher scores in applicability and importance, although the difference was statistically significant only between the iHPHs and non-iHPHs and not for the other compared pairs of hospitals.

**Figure 1:**
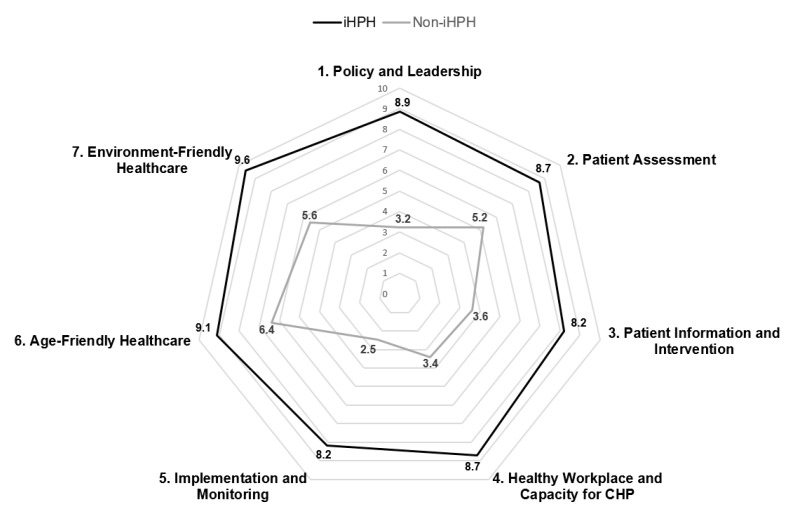
Radial plot graph of the standard compliance between iHPHs and non-iHPHs.

Figure 1 compares the standardized scores of the seven standards between the iHPHs and non-iHPHs. The iHPHs had significantly higher compliances than the non-iHPHs in all seven standards. Certificated iHPHs had the highest compliance scores in Standard 7 (environment-friendly healthcare) (9.6) and the lowest in Standards 3 (patient information and intervention) (8.2) and 5 (implementation and monitoring) (8.2). The non-iHPHs had the highest compliance score in Standard 6 (age-friendly healthcare) (6.4) and lowest in Standard 5 (implementation and monitoring) (2.5). The most prominent differences between the iHPHs and the non-iHPHs were found in Standards 1 (policy and leadership) and 5 (implementation and monitoring), and a lesser difference was found in Standard 6 (age-friendly healthcare).

### 3.4. Specialists’ Evaluation

According to the evaluation carried out by a group of specialists convened by this study, we further assessed measurable elements for comprehensibility, applicability, and importance. More than 80% of the respondents strongly agreed or agreed on the comprehensibility of the sub-standards and more than 70% of respondents strongly agreed or agreed on the applicability and importance of the sub-standards. With regard to applicability, sub-standards 5.1.4 (70%), 5.2.1 (74%), 5.2.2 (72%), and 5.2.3 (70%) received slightly less agreement. With regard to importance, sub-standards 5.2.3 (70%) and 5.2.4 (70%) received slightly less agreement. According to the findings from narrative feedback, small or district hospitals found difficulties incorporating health promotion services into their operating procedures (sub-standard 5.1.4) and conducting satisfaction surveys of information for patients (sub-standard 5.2.4); they recommended access to national clinical guidelines or pathways. One children’s hospital expressed difficulties in committing to the prevention of tobacco or betel nuts, which is a required item in the tool but not a highly relevant concern for a children’s hospital. In the research and development of CHP (sub-standard 5.2.3), six small hospitals expressed a lack of confidence in their research capacity.

## 4. Discussion

Taiwan’s HPA has developed a synergetic model for implementing health promotion in hospitals. In this study, we have assessed the validity of an integrative self-assessment tool in support of this model. This synergetic model is expected to be a parsimonious and efficient approach to implementing health promotion. By conducting EFA via stratification; testing the internal consistency, content, and construct validity; and convening a group of specialists to evaluate the self-assessment tool, our study concludes that the tool is valid and can be used in implementing such a synergetic model.

According to the EFA via stratification, each standard contained exactly one factor with four to eleven measures, which suggests that our data are suitable for factor analysis and sound factor structures. The variable-to-factor ratios were more than four, which conformed to at least four variables per factor suggested in previous studies [34,35]. The KMO values were larger than the recommended value of 0.5, and the Bartlett’s test of sphericity was statistically significant. Our study, therefore, suggests the factorability of the data [36]. No item required removal from each standard because the factor loadings of all items were larger than 0.4 [37,38]. The variances explained were larger than the acceptable threshold of 0.5 [39]. The Cronbach’s alpha coefficients for the seven dimensions of the assessment tool were all higher than 0.7, which is beyond the minimum score for adequate reliability [40]. Sample size is important for valid EFA but it is not the sole criteria [34,41]. The recommended sample-to-variable ratios range from 3:1 [42] and 5:1 [43] to 20:1 [44]. Guadagnoli and Velicer have argued that factor analysis should also emphasize the absolute magnitude of the loadings and number of variables per factor in addition to sample size [45]. The authors have further suggested that factors with four or more loadings above 0.60 in absolute value are reliable regardless of the sample size. Another study has also demonstrated that an EFA with a small sample size of below 50 may yield reliable results under the conditions of high levels of loadings, a low number of factors, and a high number of variables [41]. In this study, the sample-to-factor ratios of the seven standards were larger than the suggested threshold of 3:1, and except for Standard 3, the sample-to-factor ratios were larger than the suggested threshold of 5:1. Overall, in this study, only one factor was extracted from each standard, loadings for each factor were above 0.60, and the factors contained four to eleven variables. The factors in this study are therefore reliable in spite of the relatively small sample size.

The ceiling effect is said to occur when participants’ scores cluster toward the high end (or best possible score) of the measure/instrument; the opposite is the floor effect [46]. Floor and ceiling effects are present when at least 15% of respondents score the lowest or highest possible score, respectively [47]. This study found weak floor and mild ceiling effects, which can be visually verified by the left-skewed distribution of the overall compliance scores of the iHPH standards, indicating that a relatively large number of hospitals could achieve full or close to full scores in many of the standards or sub-standards. From a longitudinal perspective, the problems of ceiling effects may be additively increasing because participants will probably be improving on the tests as time passes; more participants will also reach the ceiling at a certain point during the observed period [48]. The relatively high ceiling effect found in this study might be explained by the time that had elapsed following the implementation of certifications of some sort. That is, HPH, age-friendly healthcare, environment-friendly healthcare, and smoke-free hospital initiatives had been in place for years in Taiwan by the time of the study. Nearly more than half of hospitals in the study had been certificated as HPHs (54.3%), age-friendly healthcare hospitals (50.0%), smoke-free hospitals (71.7%), or environment-friendly hospitals (56.5%), or had received international HPH membership (47.8%), before 2016. In Groene’s study, hospitals that were members of the HPH network were able to reach significantly higher levels of compliance with HPH standards. The present study also demonstrates that hospitals with previous certifications or membership can achieve high levels of compliance with individual and overall iHPH standards. By contrast, minor floor effects might be associated with hospital size. Half of the hospitals in this study were equipped with less than 300 beds. Among those with the lowest compliance, that is, those that failed to achieve all measurable actions in Standard 5 (a failure rate of 100% or 10/10), were those with less than 300 beds. This finding might be associated with the economy of scale in implementing health promotion in hospitals. A recent review has found consistent evidence of the economy of scale for hospitals with 200–300 beds [49]. In our study, approximately 35% of the hospitals had less than 200 beds and the compliance scores might have been compromised by these hospitals without the benefit of economy of scale.

The implementation of health promotion in hospitals worldwide still has room for improvement and leaves some undesirable gaps to be filled. Examples include those from the following list. Unmet information needs have been found in newly diagnosed breast cancer patients [50] and unmet dietary information has been found among colorectal cancer survivors [51]. Systematic reviews have found that high prevalence estimates of work-related musculoskeletal disorders have been found among surgeons [52] and that a high level of burnout cases have been found among ICU professionals [53]. These incidents might be attributed to the fact that health promotion had been implemented on an ad hoc basis or in an unsystematic manner and had not been integrated into quality MS or received adequate organizational support. The iHPH self-assessment tool is meant to address such problems and assist hospitals in building the capacity to implement multi-pronged health promotion in a systematic way and to achieve optimal health outcomes. Organizational capacity building for health promotion in structure and process could contribute to a desirable outcome in light of Donabedian’s structure–process–outcome conceptual framework [54]. Facilitated by the iHPH self-assessment tool, hospitals could create a comprehensive cross-disciplinary platform with which to coordinate relevant health promotion tasks, build supportive infrastructure, and develop routine operations to achieve optimal health outcomes. 

Regarding standardized compliance with the iHPH standards, the iHPHs had significantly higher compliances than the non-iHPHs across all seven standards. The most prominent differences between the iHPHs and non-iHPHs were found in Standards 1 (policy and leadership) and 5 (implementation and monitoring). A study in Iran has found a similar pattern of differences [55]. Standards 1 and 5 are related to the essential organizational capacity for HPH. Standard 1 focuses on CHP, continuity, and coordination of patient care procedures in hospitals, while Standard 5 is concerned with the availability of the coordinating personnel, budget, space/facilities, and health promotion services in operating procedures and a mechanism to monitor CHP performance. The gaping differences in these two standards may be explained by the fact that iHPH certification will exert extra pressure on the higher managerial echelon of the hospital. The latter will push them to be more assertive in pursuing renovated policies, including providing staff members with the necessary resources for the implementation of iHPH in accordance with the explicit standards of the certification requirements.

The current iHPH self-assessment tool was meant for hospitals of all sizes and types; however, some of the hospitals will want to see added flexibility in the tool. In Taiwan, 62% of hospitals have been equipped with less than 200 beds and have had no benefit of the economy of scale. Small hospitals have tended to have difficulties in affording the development and implementation of health-promotion-including operating procedures and expending their limited resources on satisfaction surveys of health education for patients. The prevalence of smoking and betel nut consumption among adults was 15.3% and 8.4%, respectively, in 2016 in Taiwan [19]. Quitting tobacco and betel nuts was a required measure in the tool. However, only around 3.3% of district hospitals (12/361) provided betel quid cessation programs in 2018 [56]. Further support for the development of national clinical guidelines, personnel training in quitting betel nuts, and incentives for betel quid cessation programs are inadequate. Certain flexibility must be allowed in the self-assessment tool. For example, tobacco and betel nut consumption is not the main health problem in children’s hospitals, and one must recognize that not every hospital has the capacity for research. 

According to our analysis, this self-assessment tool for health promotion in hospitals can serve as a reference for other countries with hospitals committed to health promotion. Hospitals over the globe encounter comparable challenges: non-communicable diseases account for a large proportion of deaths, which are mostly related to behavioral risk factors, including harmful use of alcohol and tobacco, dietary behavior, and physical inactivity [11]. Across the globe, populations aged 60 and over are growing faster than those for all younger age groups, with the elderly population accounting for 13% of the global population, 25% of the European population, and 22% of the North American population [57]. Consequently, age-friendly healthcare has become imperative [58,59]. Limited health literacy is a problem on a global scale and achieves rates of 47% in Europe [60] and 55% in Southeast Asian [61]. Limited health literacy has contributed to sub-optimal healthcare outcomes [62,63]. Health-literate organizations and environments can enable access to easy-to-understand health information [30]. Moreover, SDM has not been widely adopted owing to a lack of systematic promotion at national, regional, or organizational levels [64,65]. Furthermore, healthcare sectors are one of the major contributors to carbon footprints. They made up 7% of the carbon footprint in Australia (2014–2015) [66] and 10% in the USA (2016) [67]. Separately dealing with these many challenges is less effective for hospitals [22]. Thus, an integrative self-assessment tool is called for to lend itself to hospitals in synergistically responding to various health needs.

Two major limitations must be acknowledged for this study. Given the use of a self-assessment tool, the respondents may have had an incentive to overstate their performance to preserve the reputation of their institutions. Although this problem is shared by studies using a similar approach, we made efforts to address this potential issue. When distributing the questionnaire, we emphasized that the study was solely aimed at the improvement of the self-assessment tool and had no bearing on the certification itself. This tool has only been validated for Taiwan and further validation in other countries is needed if the tool is to be adopted internationally.

## 5. Conclusions

This study has presented an integrative HPH model to redress the difficulties involved in simultaneously administering a plural system of multiple certifications. Our assessment of factor analysis, internal consistency, content and construct validity, and specialists’ evaluation has suggested that the integrative self-assessment tool is valid. iHPHs exhibited significantly higher compliance rates than non-iHPHs across the seven standards. iHPHs had the highest compliance score for environment-friendly healthcare and the lowest compliance score for standards regarding implementation and monitoring as well as those regarding patient information and intervention. The most prominent differences between the iHPHs and the non-iHPHs were found in the standards on policy and leadership and on implementation and monitoring. The integrative self-assessment tool may provide a reference for international hospitals that commit to implementing multi-pronged health promotion in a world of multi-faceted challenges.

## Figures and Tables

**Table 1:** Assessed compliance of the tool.

Standards and Sub-Standards	Completely Fulfilled *n* (%)	Partly Fulfilled *n* (%)	Not Fulfilled *n* (%)
Standard 1: Policy and Leadership (0–14)			
1.1.1 The hospital has a clinial health promotion (CHP) policy which encompasses patients, family members, communities, and staff.	30 (65.2)	5 (10.9)	11 (23.9)
1.1.2 Hospital staff are involved in the formulation, auditing, and review of the policy.	31 (67.4)	3 (6.5)	12 (26.1)
1.1.3 The hospital includes CHP in its current quality and management plans.	23 (50.0)	7 (15.2)	16 (34.8)
1.1.4 The hospital prohibits the acceptance of donations and/or sponsorships from tobacco vendors and the sales of tobacco or e-cigarette products.	37 (80.4)	0	9 (19.6)
1.2.1 Hospital executives value the health plans and demands of the surrounding communities and are involved in interdepartmental and intradepartmental collaboration projects.	33 (71.7)	3 (6.5)	10 (21.7)
1.2.2 The hospital can provide a roster of health and social care resources and partners.	29 (63.0)	2 (4.3)	15 (32.6)
1.2.3 The hospital has written cooperation plans with its healthcare partners to improve the continuity of patient care.	30 (65.2)	7 (15.2)	9 (19.6)
Standard 2: Patient Assessment (0–10)			
2.1.1 The hospital has clinical guidelines and procedures for identifying smoking/betel nut consumption/alcohol consumption histories, activity levels, the nutritional status, and the psycho-socioeconomic status of patients on their first admission or visit. These guidelines or procedures are implemented and reviewed/amended annually.	29 (63.0)	8 (17.4)	9 (19.6)
2.1.2 The assessment of patients’ health promotion needs is written in their medical records.	34 (73.9)	0	12 (26.1)
2.1.3 Patients’ sociocultural preferences are detailed in their medical records to facilitate the provision of specialized care.	33 (71.7)	7 (15.2)	6 (13.0)
2.1.4 Patients’ referring physicians or other relevant sources are detailed in their medical records.	37 (80.4)	3 (6.5)	6 (13.0)
2.2.1 The hospital has clinical guidelines or procedures for reassessing patients’ health promotion needs at discharge or at the end of clinical intervention. These guidelines or procedures are reviewed/amended and improvement measures implemented annually.	27 (58.7)	9 (19.6)	10 (21.7)
Standard 3: Patient Information and Intervention (0–22)			
3.1.1 General health information and information concerning high-risk diseases are provided to the patient.	37 (80.4)	3 (6.5)	6 (13.0)
3.1.2 Information regarding patients’ self-supporting organizations is available.	27 (58.7)	5 (10.9)	14 (30.4)
3.1.3 Work protocols (procedures and guidelines) developed by multidisciplinary teams are in place.	30 (65.2)	3 (6.5)	13 (28.3)
3.1.4 The hospital has clinical guidelines or procedures for providing information, suggestions, and preliminary intervention services or measures for particular health issues (e.g., smoking, betel nut consumption, alcohol consumption, physical activity, nutrition, and psycho-socioeconomic problems). These guidelines or procedures are reviewed/amended and improvement measures implemented annually.	27 (58.7)	8 (17.4)	11 (23.9)
3.1.5 Health promotion information and services provided to patients are documented in their medical records.	26 (56.5)	8 (17.4)	12 (26.1)
3.1.6 Health promotion activities, intervention services, rehabilitation/follow-up treatment provided to patients, expected outcomes, and evaluations are documented in their medical records.	27 (58.7)	6 (13.0)	13 (28.3)
3.1.7 The hospital promotes a shared decision-making (SDM) plan and provides a favorable communication environment for patients and their family members in which to obtain information, thereby fostering their ability and safeguarding their right to make decisions concerning care services.	19 (41.3)	7 (15.2)	20 (43.5)
3.2.1 The hospital has clinical guidelines or procedures for the provision of intensive intervention services, rehabilitation, or treatment for particular issues (e.g., smoking, betel nut consumption, alcohol consumption, physical activity, nutrition, and psycho-socioeconomic problems). These guidelines or procedures are reviewed/amended and improvement measures implemented annually.	25 (54.3)	4 (8.7)	17 (37.0)
3.2.2 Patients (or their family members) are provided with easy-to-understand follow-up advice at outpatient visits, referrals, or upon discharge.	32 (69.6)	6 (13.0)	8 (17.4)
3.2.3 The receiving hospital promptly provides patients with written summaries concerning their conditions, health needs, and intervention and clearly defines its role and the roles of its partners in their medical records (e.g., with a rehabilitation plan).	31 (67.4)	3 (6.5)	12 (26.1)
3.2.4 The hospital has a health-literacy-promoting plan that aims to help patients obtain, comprehend, and apply information and services to improve their health and the provision of care.	25 (54.3)	14 (30.4)	7 (15.2)
Standard 4: Promoting a Healthy Workplace and Ensuring Capacity for CHP (0–8)			
4.1.1 Staff comply with health and safety requirements and all risk factors in the workplace are identified.	31 (67.4)	6 (13.0)	9 (19.6)
4.1.2 Staff have health promotion options, including smoking cessation, betel nut cessation, alcohol abstinence, nutrition, vaccinations, mental health in the workplace, and physical activities.	33 (71.7)	6 (13.0)	7 (15.2)
4.1.3 Annual staff surveys are conducted. Survey content should encompass assessments of personal health, understanding of relevant services and policies, and utilization of health promotion activities.	25 (54.3)	7 (15.2)	14 (30.4)
4.2.1 Staff are offered CHP training and professional development programs.	28 (60.9)	4 (8.7)	14 (30.4)
Standard 5: Implementation and Monitoring (0–16)			
5.1.1 The hospital has designated staff member(s) responsible for coordinating health promotion activities.	31 (67.4)	2 (4.3)	13 (28.3)
5.1.2 The hospital has a budget for funding health promotion services.	30 (65.2)	3 (6.5)	13 (28.3)
5.1.3 The hospital has space or facilities (i.e., resources, space, and equipment) for accommodating health promotion.	27 (58.7)	6 (13.0)	13 (28.3)
5.1.4 The hospital includes health promotion services in its operating procedures (e.g., clinical guidelines or pathways) and these are available in all clinical departments.	25 (54.3)	2 (4.3)	19 (41.3)
5.2.1 The hospital routinely collects health promotion intervention information and makes it available to staff for evaluation.	30 (65.2)	3 (6.5)	13 (28.3)
5.2.2 The hospital has a quality control protocol for organizing health promotion activities.	25 (54.3)	6 (13.0)	15 (32.6)
5.2.3 The hospital is involved in the research and development of health promotion.	24 (52.2)	4 (8.7)	18 (39.1)
5.2.4 The hospital performs satisfaction surveys on the information it provides to its patients and uses feedback to improve its quality management system.	26 (56.5)	3 (6.5)	17 (37.0)
Standard 6: Age-Friendly Healthcare (0–14)			
6.1.1 Accessible facilities are available for people with mobility restrictions.	32 (69.6)	12 (26.1)	2 (4.3)
6.1.2 Environments adopt universal designs.	36 (78.3)	7 (15.2)	3 (6.5)
6.1.3 A healthy environment that takes into account the physical and mental impairments of elderly patients is established.	32 (69.6)	11 (23.9)	3 (6.5)
6.2.1 Administrative procedures are adjusted to take into account the special needs of the elderly (including patients and family members).	30 (65.2)	11 (23.9)	5 (10.9)
6.2.2 A favorable communication environment is established so that elderly patients and relatives can obtain information, thereby ensuring that older adults have the ability and the right to make their own medical decisions.	30 (65.2)	11 (23.9)	5 (10.9)
6.2.3 Assistance is provided to elders with financial difficulties, or referrals are made so that elders (patients and family members) can receive suitable medical/care records and follow-up services.	38 (82.6)	5 (10.9)	3 (6.5)
6.2.4 A volunteer plan is available and effectively implemented to assist elders.	34 (73.9)	8 (17.4)	4 (8.7)
Standard 7: Environment-Friendly Healthcare (0–8)			
7.1.1 Plans and records on annual energy and water conservation plans are available.	37 (80.4)	7 (15.2)	2 (4.3)
7.1.2 Plans and records on annual medical waste reduction plans are available.	36 (78.3)	8 (17.4)	2 (4.3)
7.1.3 Plans and records on annual green procurement plans are available.	31 (67.4)	7 (15.2)	8 (17.4)
7.1.4 The hospital periodically reviews its progress and proposes improvement plans.	34 (73.9)	7 (15.2)	5 (10.9)

**Table 2:** Characteristics of participating hospitals.

Characteristics	Frequency (%)
Ownership	
Public	12 (26.1%)
Private	16 (34.8%)
Private non-profit	18 (39.1%)
Hospital level	
Medical centers	6 (13.0%)
Regional hospitals	18 (39.1%)
District hospitals	22 (47.8%)
Specialized hospital	
Yes	6 (13.0%)
No	40 (87.0%)
Number of beds	
≤100 beds	10 (21.7%)
101–300 beds	13 (28.3%)
301–600 beds	9 (19.6%)
601–1000 beds	8 (17.4%)
>1000 beds	6 (13.0%)
International membership of health-promoting hospital (HPH)	
Yes	22 (47.8%)
No	24 (52.2%)
Certificated as HPH in Taiwan	
Yes	25 (54.3%)
No	21 (45.7%)
Certificated as age-friendly healthcare	
Yes	23 (50.0%)
No	23 (50.0%)
Certificated as smoke-free hospital	
Yes	33 (71.7%)
No	13 (28.3%)
Certificated with environment-friendly healthcare	
Yes	26 (56.5%)
No	20 (43.5%)

**Table 3:** Summary of the results from exploratory factor analyses (EFA) by stratification.

Standards and Sub-Standards	Factor Loadings	Communalities		Scree Tests
	Factor 1	Initial	Extraction	
Standard 1: Policy and Leadership (0–14) Kaiser–Meyer–Olkin (KMO) = 0.886, Bartlett test: *p* < 0.001, % of variance explained = 71.932, Cronbach’s alpha = 0.944				
1.1.1	0.772	0.653	0.596	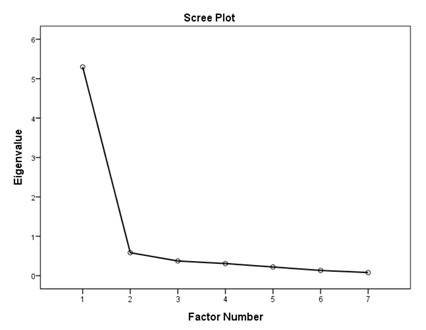
1.1.2	0.917	0.828	0.841	
1.1.3	0.829	0.723	0.687	
1.1.4	0.827	0.744	0.684	
1.2.1	0.957	0.883	0.917	
1.2.2	0.739	0.578	0.546	
1.2.3	0.875	0.803	0.766	
Standard 2: Patient Assessment (0–10) KMO = 0.801, Bartlett test: *p* < 0.001, % of variance explained = 60.487, Cronbach’s alpha = 0.878				
2.1.1	0.961	0.806	0.923	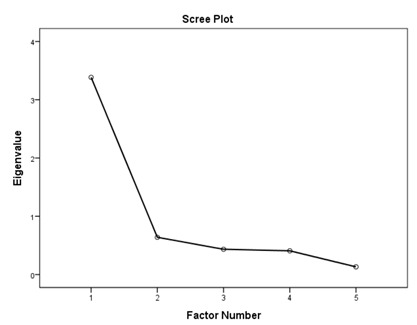
2.1.2	0.761	0.679	0.578	
2.1.3	0.704	0.462	0.496	
2.1.4	0.663	0.443	0.439	
2.2.1	0.767	0.544	0.588	
Standard 3: Patient Information and Intervention (0–22) KMO = 0.902, Bartlett test: *p* < 0.001, % of variance explained = 70.214, Cronbach’s alpha = 0.961				
3.1.1	0.707	0.585	0.499	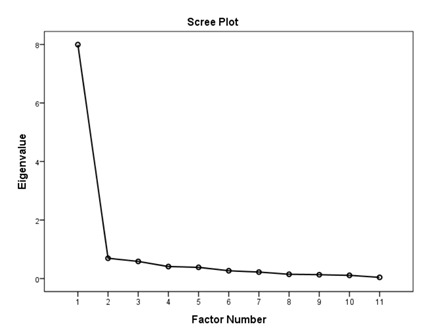
3.1.2	0.634	0.504	0.402	
3.1.3	0.922	0.915	0.849	
3.1.4	0.919	0.838	0.844	
3.1.5	0.809	0.713	0.655	
3.1.6	0.893	0.881	0.797	
3.1.7	0.787	0.739	0.619	
3.2.1	0.906	0.899	0.821	
3.2.2	0.867	0.834	0.752	
3.2.3	0.845	0.771	0.714	
3.2.4	0.878	0.806	0.772	
Standard 4: Promoting a Healthy Workplace and Ensuring Capacity for CHP (0–8) KMO = 0.812, Bartlett test: *p* < 0.001, % of variance explained = 66.991, Cronbach’s alpha = 0.887				
4.1.1	0.790	0.574	0.625	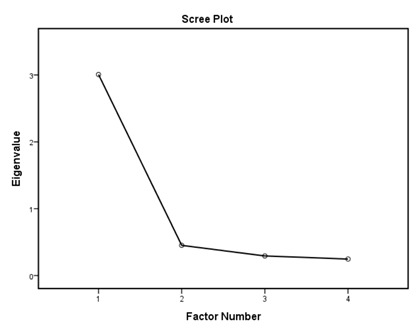
4.1.2	0.843	0.618	0.711	
4.1.3	0.783	0.567	0.612	
4.2.1	0.855	0.633	0.732	
Standard 5: Implementation and Monitoring (0–16) KMO = 0.896, Bartlett test: *p* < 0.001, % of variance explained = 75.621, Cronbach’s alpha = 0.958				
5.1.1	0.955	0.936	0.912	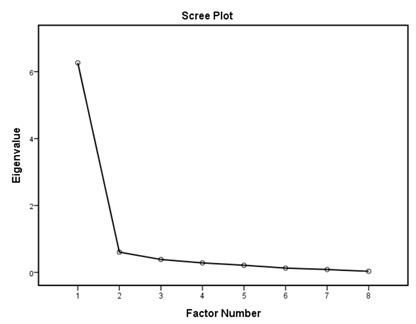
5.1.2	0.898	0.861	0.806	
5.1.3	0.903	0.841	0.815	
5.1.4	0.809	0.684	0.654	
5.2.1	0.968	0.947	0.937	
5.2.2	0.861	0.844	0.741	
5.2.3	0.637	0.438	0.406	
5.2.4	0.882	0.754	0.779	
Standard 6: Age-Friendly Healthcare (0–14) KMO = 0.807, Bartlett test: *p* < 0.001, % of variance explained = 55.780, Cronbach’s alpha = 0.896				
6.1.1	0.623	0.543	0.388	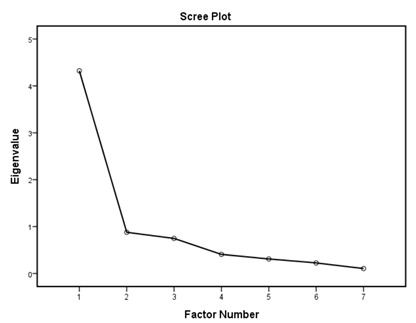
6.1.2	0.707	0.582	0.500	
6.1.3	0.688	0.565	0.473	
6.2.1	0.829	0.802	0.687	
6.2.2	0.882	0.816	0.777	
6.2.3	0.788	0.644	0.620	
6.2.3	0.677	0.519	0.458	
Standard 7: Environment-Friendly Healthcare (0–8) KMO = 0.767, Bartlett test: *p* < 0.001, % of variance explained = 80.289, Cronbach’s alpha = 0.923				
7.1.1	0.969	0.941	0.939	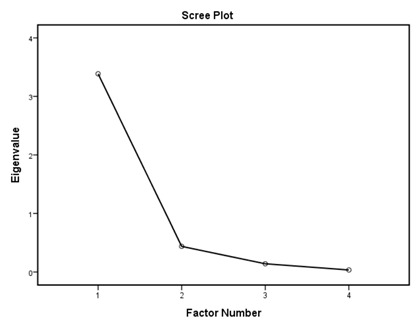
7.1.2	0.925	0.925	0.855	
7.1.3	0.739	0.602	0.546	
7.1.4	0.933	0.821	0.871	

**Table 4:** Compliance with standards: distribution, floor, and ceiling effects.

	Standard 1: Policy and Leadership *n* (%)	Standard 2: Patient Assessment *n* (%)	Standard 3: Patient Information and Intervention *n* (%)	Standard 4: Healthy Workplace and Capacity for CHP *n* (%)	Standard 5: Implementation and Monitoring *n* (%)	Standard 6: Age-Friendly Healthcare *n* (%)	Standard 7: Environment-Friendly Healthcare *n* (%)	Overall Compliance *n* (%)
Theoretical range in the compliance score	0–14	0–10	0–22	0–8	0–16	0–14	0–8	0–92
Hospitals (%) with lowest score	5 (10.9)	4 (8.7)	3 (6.5)	6 (13.0)	10 (21.7)	1 (2.2)	2 (4.3)	0
Hospitals (%) with highest score	18 (39.1)	18 (39.1)	10 (21.7)	18 (39.1)	12 (26.1)	21 (45.7)	29 (63.0)	6 (13.0)
Skewness	−1.00	–1.37	–0.78	–0.95	–0.72	–1.462	–1.65	–0.94
Kurtosis	–0.63	0.62	–1.04	–0.66	–1.28	1.596	1.66	–0.77
Observed compliance score: mean (SD); median	9.85 (5.2) 13.0	7.54 (3.2) 9.0	14.76 (8.0) 19.0	5.59 (2.9) 7.0	10.11 (6.5) 14.0	11.50 (3.4) 13.0	6.63 (2.3) 8.0	60.0 (29.5) 80.5

**Table 5:** Compliance and assessment of measurable elements by hospital characteristics.

			Mean (SD) Self-Reported Level of Compliance with Integration of HPH (iHPH) Standards								Mean (SD) Assessment of Measurable Elements	
		*n*	Policy and Leadership	Patient Assessment	Patient Information and Intervention	Healthy Workplace and Capacity for CHP	Implementation and Monitoring	Age-Friendly Healthcare	Environment-Friendly Healthcare	Overall Compliance	Applicability	Importance
iHPH	Yes	31	12.42 (3.05)	8.68 (2.29)	18.06 (5.29)	6.97 (1.70)	13.10 (4.22)	12.74 (1.93)	7.68 (0.83)	79.65 (17.23)	165.61 (20.47)	167.48 (20.61)
	No	15	4.53 (4.85)	5.20 (3.73)	7.93 (8.48)	2.73 (2.89)	3.93 (6.17)	8.93 (4.37)	4.47 (2.85)	37.73 (29.99)	151.60 (20.09)	155.93 (21.45)
			*p* < 0.001	*p* = 0.001	*p* = 0.001	*p* < 0.001	*p* < 0.001	*p* = 0.003	*p* < 0.001	*p* < 0.001	*p* = 0.024	*p* = 0.059
HPH	Yes	25	12.24 (3.35)	8.64 (2.48)	18.08 (5.46)	7.00 (1.55)	12.92 (4.54)	12.68 (1.95)	7.72 (0.84)	79.28 (17.98)	163.64 (20.46)	165.00 (20.85)
	No	21	7.00 (5.73)	6.24 (3.60)	10.81 (8.84)	3.90 (3.30)	6.76 (7.03)	10.10 (4.23)	5.33 (2.80)	50.14 (33.02)	157.95 (22.13)	162.19 (22.37)
			*p* = 0.001	*p* = 0.009	*p* = 0.008	*p* = 0.002	*p* = 0.001	*p* = 0.053	*p* < 0.001	*p* = 0.005	*p* = 0.365	*p* = 0.574
International HPH Network Member	Yes	22	12.17 (3.40)	8.67 (2.53)	18.08 (5.58)	7.00 (1.59)	13.04 (4.59)	12.71 (1.13)	7.75 (0.85)	79.42 (18.36)	164.54 (20.39)	166.00 (20.68)
	No	24	7.32 (5.79)	6.32 (3.54)	11.14 (8.76)	4.05 (3.29)	6.91 (6.90)	10.18 (1.73)	5.41 (2.75)	51.32 (32.70)	157.23 (21.86)	161.23 (22.29)
			*p* = 0.003	*p* = 0.006	*p* = 0.007	*p* = 0.002	*p* = 0.001	*p* = 0.039	*p* < 0.001	*p* = 0.003	*p* = 0.239	*p* = 0.367
Age-Friendly Healthcare	Yes	23	12.65 (1.67)	9.35 (0.89)	19.57 (3.09)	7.26 (1.05)	13.83 (3.13)	13.13 (1.58)	7.91 (0.29)	83.70 (8.05)	166.35 (20.17)	167.13 (20.30)
	No	23	7.04 (6.09)	5.74 (3.73)	9.96 (8.56)	3.91 (3.25)	6.39 (6.96)	9.87 (3.98)	5.35 (2.71)	48.26 (32.61)	155.74 (21.28)	160.30 (22.29)
			*p* = 0.007	*p* < 0.001	*p* < 0.001	*p* < 0.001	*p* = 0.001	*p* = 0.001	*p* < 0.001	*p* = 0.001	*p* = 0.080	*p* = 0.248
Environment-Friendly Healthcare	Yes	26	11.92 (3.27)	8.69 (2.43)	18.27 (5.40)	6.88 (1.63)	13.04 (4.38)	12.92 (1.92)	7.77 (0.82)	79.50 (17.59)	164.27 (19.55)	166.42 (19.59)
	No	20	7.15 (6.12)	6.05 (3.61)	10.20 (8.64)	3.90 (3.39)	6.30 (6.97)	9.65 (4.06)	5.15 (2.76)	48.40 (32.92)	156.85 (22.98)	160.20 (23.51)
			*p* = 0.021	*p* = 0.005	*p* = 0.002	*p* = 0.003	*p* = 0.001	*p* = 0.002	*p* < 0.001	*p* = 0.003	*p* = 0.267	*p* = 0.399
Smoke-Free Hospitals	Yes	33	11.70 (3.77)	8.61 (2.50)	17.97 (5.46)	6.76 (1.79)	12.36 (5.11)	12.82 (1.79)	7.55 (1.20)	77.76 (19.20)	163.97 (19.51)	166.33 (19.32)
	No	13	5.15 (5.66)	4.85 (3.44)	6.62 (7.78)	2.62 (3.20)	4.38 (6.37)	8.15 (4.28)	4.31 (2.81)	36.08 (30.64)	153.62 (24.21)	157.08 (25.48)
			*p* < 0.001	*p* < 0.001	*p* < 0.001	*p* < 0.001	*p* = 0.001	*p* = 0.001	*p* < 0.001	*p* < 0.001	*p* = 0.168	*p* = 0.213
Bed Numbers	≤300	23	7.48 (5.88)	6.04 (3.51)	10.39 (8.35)	4.09 (3.32)	6.70 (6.77)	9.96 (3.91)	5.52 (2.75)	50.17 (31.80)	154.70 (21.82)	159.13 (23.53)
	>300	23	12.22 (3.15)	9.04 (2.12)	19.13 (4.62)	7.09 (1.35)	13.52 (4.12)	13.04 (1.89)	7.74 (.86)	81.78 (15.85)	167.39 (18.93)	168.30 (18.32)
			*p* = 0.008	*p* = 0.001	*p* < 0.001	*p* = 0.002	*p* < 0.001	*p* = 0.001	*p* = 0.002	*p* = 0.001	*p* = 0.057	*p* = 0.159
